# Rare neonatal diabetes insipidus and associated late risks: Case report

**DOI:** 10.1186/1471-2431-12-56

**Published:** 2012-05-28

**Authors:** Maximiliano Francisco Rivas-Crespo, Lorena Miñones-Suárez, Susana Serrano G-Gallarza

**Affiliations:** 1 Department of Pediatrics, Hospital Universitario Central de Asturias, Oviedo, Spain; 2 Neuroradiology, Hospital San Agustín, Avilés, Spain

**Keywords:** At term newborn, Infant, Coarctation of the aorta, Neonatal intraventricular hemorrhage, Central diabetes insipidus, Neurodevelopmental delay, Undernutrition, Myelinolysis, Dehydration, Desmopressin

## Abstract

**Background:**

Most cases of neonatal central diabetes insipidus are caused by an injury, which often results in other handicaps in the patient. The infant’s prognosis will be determined by his or her own early age and disability as well as by the physician’s skill. However, the rarity of this condition prevents the acquisition of personal experience dealing with it.

**Case Presentation:**

A neonatal hemorrhagic stroke, caused by an aortic coarctation, caused right lower limb paresis, swallowing disability, and central diabetes insipidus in a term infant. The scant oral intake, as a consequence of his disability, caused progressive undernutrition which closed a vicious circle, delaying his development and his ability to overcome the swallowing handicap. On the other hand, nasal desmopressin absorption was blocked by several common colds, resulting in brain bleeding because of severe dehydration. This even greater brain damage hampered the improvement of swallowing, closing a second harmful circle. Moreover, a devastating central myelinolysis with quadriplegia, caused by an uncontrolled intravenous infusion, consummated a pernicious sequence, possibly unreported.

**Conclusions:**

The child’s overall development advanced rapidly when his nutrition was improved by gastrostomy: This was a key effect of nutrition on his highly sensitive neurodevelopment. Besides, this case shows potential risks related to intranasal desmopressin treatment in young children.

## Background

Symptomatic perinatal hemorrhagic stroke, which affects 6.2 per 100,000 live births, is highly infrequent among term babies without perinatal risks [1]; the rare cases reported have almost always been mild. Neonatal intraventricular hemorrhage (IVH) is usually related to bleeding disorders or vascular anomalies [2], coarctation of the aorta being an uncommon cause, although it can cause serious early bleeding [3].

Neonatal central diabetes insipidus (NCDI), a cause of high mortality and serious morbidity, is broadly related to hypothalamic injury. However, posthemorrhagic cases are very infrequent and only a few of these affect term infants [4]. Concurrence of all these rare conditions, concomitant in a single patient, is an exceptional didactic experience worthy of attention.

Both oral and nasal routes are accredited in desmopressin treatment for infants with NCDI [5]. However, these pathways may not be safe for these frequently disabled patients at a young age, as will be seen.

Myelinolysis constitutes a very dangerous osmotic demyelination which may result in quadriparesis or death. It is related to an abrupt change in serum osmolality, being more frequent in undernourished patients [6,7]. Its diagnosis is usually supported by magnetic resonance imaging (MRI) [8]. However, clinical and physiopathogenic evidence can identify a case of myelinolysis unnoticed by MRI, as we shall also see.

## Case Presentation

**Figure 1:**
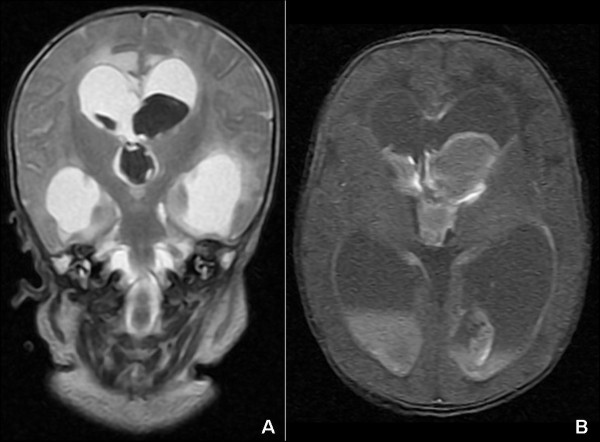
**Neonatal intraventricular hemorrhage.** MR images of subacute intraventricular hemorrhage in both lateral and third ventricles, causing intraventricular obstructive hydrocephalus. Layering fluid/fluid (fluid/heme) levels are present in occipital horns. Brainstem appears unremarkable. (Left: coronal T2WI, right: axial T1WI).

A 28-day-old baby boy, born at term from an uneventful pregnancy and vaginal delivery (Apgar score: 9/10), was referred from a nearby town to our hospital after a week of irritability and feeding difficulties. He was a 3200 g. (cephalic perimeter: 38.5 cm), phenotypically normal infant, seriously lethargic, hypertonic, with intermittent lower right limb clonic seizures. His breath was superficial, and suck, grasp and Moro reflexes were absent. His anterior fontanel was bulging, and his cerebrospinal fluid was xanthochromic. Although serum electrolytes, creatinine, C-protein and blood cell count were normal, the child presented metabolic acidosis (pH: 7.18; HCO_3_: 15.5 mmol/L). Brain MRI (Figure 1) confirmed a massive IVH with periventricular infiltration and hydrocephalus. Moreover, an II/VI systolic murmur, with upper arm blood pressure up to 178/94 mm Hg, and 57/49 mm in his leg, led to the diagnosis of aortic coarctation.

The patient’s initial course, under mechanical ventilation, captopril and phenobarbital, was favorable. Five days later, however, sudden polyuria (9 ml/kg/h), clinical signs of dehydration with unexpected weight loss (6%), and hyposthenuria (124 mOsm/kg), despite high serum sodium (156 mmol/L), chloride (126 mmol/L) and osmolality (326 mOsm/kg), led to the diagnosis of CDI. Subcutaneous (sc) desmopressin (0.02 μg. b.i.d.) achieved a rapid recovery (average serum sodium, 138 mmol/L; osmolality, 290 mOsm/kg). Surgical correction of his severe juxtaductal coarctation was performed 6 days after his metabolic stabilization. A ventriculoperitoneal shunt was performed shortly thereafter. Due to the child’s severe deglutory handicap, sc desmopressin therapy was still recommended at discharge. The family was instructed to perform subcutaneous injection and daily diuresis and body weight control at home, and the patient was scheduled for periodic clinical and analytical (serum sodium) check-ups.

Four months later, the infant maintained a proper fluid balance. However, his delayed growth and neurodevelopment was progressively more evident, and his swallowing disability had almost led to malnourishment (Table 1). He was scheduled for quarterly monitoring and nutritional support at his local hospital, under whose control he remained until two years of age. Throughout this period, his scant oral intake was supported by means of continuous debit enteral nutrition and he received desmopressin (0.1 μg bid, sc), phenobarbital (15 mg bid) and captopril (1 mg tid).

**Table 1:** Sequential events in the patient’s evolution

**Age**	**Clinical event**	**DDAVP therapy**	**Nutritional support**	**Deglutition**	**Serum**		**Length cm (SDS)**	**Weight kg (SDS)**	**C. P. cm (SDS)**
					**Sodium mmol/L**	**Osmolatity mOsm/kg**			
28 days	IVH, CoA	--		disability	136	-	53(−0.3)	3.2 (−1.8)	38.5 (1.6)
33 days	CDI (diagnosis)	0.02 μg, bid sc 10 μg			156	326		3.01 (−2.2)	
4 mo.	undernourished		oral		140	288	60(−1.2)	5.1 (−1.9)	
2 y.	undernourished failed gastrostomy dehydration episodes		CDEN		146	289	83.5(−1.7)	10.3(−1.9)	45 (−3.5)
3 y.	undernourished	bid oral			147	294	90.5(−2.1)	11.1 (−2.1)	
3 y.5 mo.	severe dehydration				189	-	-	8.3 (−3.1)	
2d. later	myelinolysis quadriplegia				147	287	-	9.4 (−2.6)	
3 y.6 mo.		0.3 μg, bid sc	gastrostomy		142	-	93 (−2.0)	10.6 (−2.7)	
4 y.	deglutition (progressively)			ability	135	279	99 (−1.14)	16.9 (−0.3)	
5 y.		0.3 μg, bid oral	--		137	280	105(−1.3)	21.2 (0.4)	46 (−4.5)

IVH: intraventricular hemorrhage. CoA: Coarctation of the aorta. CDI: Central diabetes insipidus. DDAVP: desmopressin. C.P.: cephalic perimeter. CEDEN: continuous debit enteral nutrition.

When he returned at two years of age, he had had no seizures, his ventriculoperitoneal shunt was working properly and, due to the fact that his food intake had been stable (though low), with no significant vomiting or diarrhoea, he maintained almost perfect fluid control. However, we saw a small, thin, microcephalic child (Table 1), with very small developmental progress and whose deglutition had just improved. His serum sodium (146 mmol/L), osmolality (294 mOsm/kg), glucose (4.0 mmol/L), a.m. cortisol (292.6 mmol/L), free thyroxin (19.6 pmol/L), insulin (2.2 μU/mL) and IGF-1 (78 ng/mL) were within normal range, but his vasopressin was lesser than 1.1 pmol/L. His urinary osmolality ranged from 758 mOsm/kg (3 hours after desmopressin) to 226 mOsm/kg prior to the following dose. Given his unfavourable nutritional evolution, with insufficient deglutition, a gastrostomy was advised to provide nutritional support.

At the three-year check-up, the patient was under nasal desmopressin treatment (10 μg bid). This was recommended at his local hospital some four months before, when he underwent a gastrostomy, which unfortunately soon failed because of septic complications. Subsequently, the undernourished (Table 1) and seriously delayed child (developmental age of about 9–10 months) got progressively worse: His fluid balance became quite unstable and, on at least two occasions, he suffered weight loss and clinical deterioration for several days related to a common cold. The family was informed about the risk posed by the probable dehydration episodes suffered by the child. The sc route for the desmopressin therapy and a new attempt at gastrostomy were once again recommended.

This advice was not followed, and at the age of 3 years and 5 months, the child was admitted to his local hospital after five days of a new nasal cold. His medical report registered a Glasgow Score of 8, body weight loss of 12%, and 189 mmol/L of serum sodium. After a few hours of saline infusion, he achieved a certain degree of clinical recovery. However, the next day he worsened again, and forty hours after his admittance, he entered into status epilepticus and was transferred to our hospital.

**Figure 2:**
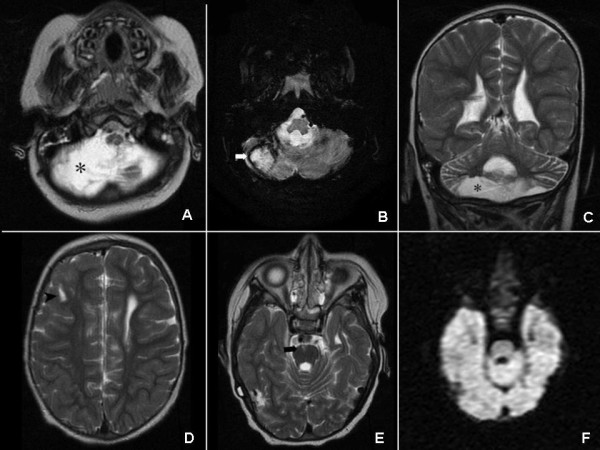
**MRI at 3 y. 4 mo. of age.** Axial spin echo T2 WI (A), axial gradient echo T2 WI (B) and coronal T2 weighted (C) sections showing chronic bilateral cerebellar haemorrhagic infarctions as encephalomalacia and loss of parenchyma (asterisks). Note hemosiderin (white arrow). D, E: Axial spin echo T2 images showing chronic-appearing lacunar infarctions in subcortical white matter of the right frontal lobe (short black arrow) and peripheral pons (black arrow). No osmotic demyelination features in central pontine fibers. F: Axial diffusion weighted image reveals no restricted diffusion in central pons.

We received a sedated, malnourished child, with an irregular breathing rate and whose serum sodium was 147 mmol/L. Electroencephalogram and brain CT confirmed the suspected cerebral edema. After two weeks of intensive care, he was fully awake, but was quadriplegic, with hyperreflexic, spastic limbs, wrists in palmar flexion, bilateral Babinski sign, and a severely hypotonic neck. Central pontine myelinolysis was diagnosed; however, MRI were not those expected for this diagnosis, but rather of old hemorrhagic infarcts (Figure 2). On discharge, desmopressin (0.13 μg bid, sc) was once again recommended and phenobarbital was replaced by levetiracetam (200 mg bid). Gastrostomy was uneventfully restored two weeks later.

Six months later (four years of age), sc desmopressin did maintain a fair balance. The deglutition ability of our quadriplegic patient had progressed admirably and his nutrition was greatly improved (Table 1). His facial expression and babbling likewise evidenced some developmental improvement.

Despite his severe cognitive impairment, over the following year our patient improved significantly in terms of his cervical control, a useful ability to push with his left hand likewise being perceived. Poor, but increasingly disyllabic verbal expression and a significant capacity to understand simple verbal messages were also appreciated. As he had achieved an almost normal swallowing ability, his gastrostomy was closed and desmopressin was returned to the oral route (0.3 -0.35 μg bid).

At the check-up some days before his fifth birthday, he was an overweight boy (Table 1).

## Discussion

Aortic coarctation may be associated with several other cardiac or vascular anomalies and may be part of various syndromes, such as those of Turner, Noonan, Kabuki, PHACE, or Alagille; none of these conditions was identified in our patient.

Intraventricular hemorrhage in at-term newborns are infrequent and mostly mild, but those especially rare cases related to aortic coarctation, such as that of our patient, involve severe bleeding, usually in the first days of life [2,3]. Despite the severity of his condition, his clinical onset was late and subacute, a fact that possibly led to a harmful delay in medical assistance.

Periventricular white matter damage produced by hydrocephalic IVH may cause NCDI, although it is uncommon in term infants because of the rarity of this complication among these patients [4,9].

The rarity and high neonatal mortality of NCDI [4] explains the usually poor experience in its long-term care. The route of desmopressin administration to these often handicapped infants is an important issue. The oral route is a common alternative to the often preferred intranasal route [5], although the choice should be determined by the patient’s clinical condition. In both cases, dosing is individual and highly variable over time. Besides being uncomfortable, the latter route is frequently blocked in infants and young children because of the high prevalence of colds and upper airway infections among these subjects. Each day of nasal blockage constitutes a day of accumulated risk of hypernatremic dehydration; therefore, nasal desmopressin should be seriously questioned in this age group. Moreover, the oral route, not particularly easy in a young infant, is especially unsafe if the child has swallowing disability. The speed and time of action of sc desmopressin are similar to those achieved via the intranasal route, ensuring a more effective and even safer pharmacokinetics when treating infants [10], being especially suitable for disabled infants.

Serum sodium was markedly diminished in our brain-damaged, undernourished patient (42 mmol/L in roughly 40 hours) when he was seriously hypernatremic. This is much more than the advised upper limit of 8 mEq/L/24 hours [7]. An abrupt modification of serum osmolality, usually due to the rapid correction of hyponatremia and sometimes to that of hypernatremia [6,7], can induce osmotic demyelination of regions of white and grey matter apposition, such as the pons varolii and some symmetrical cerebral and cerebellar areas. MRI can detect restricted diffusion and a low apparent diffusion coefficient, indicative of cytotoxic edema, from the day after its onset, over approximately two weeks [11]. Axial views (T1,T2) can show an abnormal triangular signal in the central pontine area, sparing the ventro-lateral pons and tegmentum, although this may take up to two weeks [8]. Unexpectedly, our patient showed no restricted diffusion areas at all (Figure 2.F), leading us to suspect that diffusion was never restricted. Conventional MRI sequences (Figure 2. A-E) showed an abnormal pontine cortico-ventral signal (though not in the central pontine area), and in the right frontal subcortical white matter (though not symmetrical). Therefore, MRI showed no recent myelinolysis, but did evidence an advanced stage of Wallerian degeneration [12]. The lack of images of myelinolysis on MRI does not rule out this diagnosis [11]. However, the measurement of cerebrospinal myelin basic proteins, which are highly elevated from early myelinolysis, would have supported it [13].

All of these images are areas of post-infarction encephalomalacia [14]. Severe hypernatremic dehydration can produce cerebral osmotic edema, causing hypoxemia and multiple foci of hemorrhagic infarction [14,15]. The images of cortical pontine encephalomalacia and the large area of cerebellar liquefaction (Figure 2) are long term consequences of the repeated hypernatremic episodes suffered by the patient.

Undernutrition was an overall determining factor for the patient’s evolution. His growth, cervical tone, deglutition, and his overall neurodevelopment clearly improved when he was able to overcome his malnutrition. His development would have probably been much better, and perhaps myelinolysis might have been avoided, if gastrostomy had worked properly in the first attempt.

## Conclusions

Our complex, unusual and atypical case reveals that:

Intranasal desmopressin may be unsafe for a handicapped infant or young child, implying a high risk of hypernatremic dehydration and cerebral hemorrhage with serious brain damage for life.

Gastrostomy was the only useful measure to restore the nutritional status of our handicapped patient. Previous conservative measures entailed the waste of precious time, delaying his recovery and exposing him to extremely serious and irreversible complications.

Recurrent episodes of hypernatremia, related to the lability of the intranasal administration of desmopressin, were responsible for several foci of cerebral infarcts (which were at advanced stages of Wallerian degeneration when identified).

A severe pontine myelinolysis of uncommon origin, the abrupt correction of hypernatremia, could not be detected by MRI, despite its severity.

The outstanding response of the patient to nutritional improvement shows the permissive role of nutrition in neurodevelopment. Likewise, the patient would not have suffered myelinolysis if he had enjoyed a satisfactory nutritional status.

Myelinolysis would probably never have occurred without the combination of malnutrition, as a predisposing factor, and the hypernatremia related to the failure of desmopressin, as the trigger.

Scarce, severe and convoluted NCDI patients should be placed under the care of a specialized team which, in addition to a neonatologist, should include an experienced pediatric neurologist, nutritionist and endocrinologist.

### Consent

Written informed consent was obtained from the parents of the patient for publication of this case report and accompanying images. A copy of the written consent is available for review by the Editor-in-Chief of this journal.

## Competing interests

The authors declare that they have no competing interests.

## Authors’ contributions

MFR-C conceived the study and drafted the manuscript. LM-S and SSG-G participated in its design and helped to draft the manuscript. All authors read and approved the final manuscript.

## Pre-publication history

The pre-publication history for this paper can be accessed here:

http://www.biomedcentral.com/1471-2431/12/56/prepub
